# Evolution of COVID-19 infection in Punjab; trends during five waves of infection in the province of Punjab

**DOI:** 10.1186/s12879-024-09157-8

**Published:** 2024-03-25

**Authors:** Hasnain Javed, Aasia Khaliq, Shaper Mirza, Rimsha Khan, Warda Fatima

**Affiliations:** 1 Provincial Public Health Reference Laboratory, Punjab AIDS Control Program, Lahore, Pakistan; 2 Department of Life Sciences, Lahore University of Management Sciences (LUMS), Lahore, Pakistan; 3 Institute of Biochemistry and Biotechnology, University of Veterinary and Animal Sciences, Lahore, Pakistan; 4 Institute of Microbiology and Molecular Genetics, University of the Punjab, Lahore, Pakistan

**Keywords:** COVID-19, Epidemiology, Incident rate, RT-PCR

## Abstract

**Background:**

Pakistan witnessed five waves of COVID-19 infections during the pandemic. Punjab, the largest province of Pakistan, remained the epicentre due to a high infection rate. Administrative data for five waves of the pandemic was analyzed to determine the rate of infections and the significance of pharmacological and non-pharmacological interventions on the severity and duration of infection.

**Methodology:**

COVID-19 data from March 2020 to May 2023 was obtained from the Provincial Public Health Reference Laboratory (PPHRL), Punjab AIDS Control Program, Lahore. The data included samples from index cases, contacts, and recovered patients. A total of 36,252,48 cases were screened for COVID-19, and 90,923 (2.50%) were detected positive by RT-PCR, accounting for 5.69% of the cases reported positive throughout the country.

**Results:**

Among the positive cases, 50.86% (*n* = 46,244) cases were new cases (registered for the first time), 40.41% (*n* = 36751) were the contact cases traced from the newly identified cases and 8.62% (*n* = 7842) repeated cases. The positivity rates among index cases were reported to be 2.37%, 2.34%, 4.61%, 2.09%, and 1.19%, respectively, for the five respective COVID-19 pandemic waves. Distribution by gender indicated that 64% of males and 35% of females were infected during the pandemic. The age factor demonstrated the most susceptibility to infection in women aged 19-29 years, whereas most males between the ages of 29-39 had an infection. Susceptibility to COVID-19 infection was observed to be equally likely between males and females; however, clinical outcomes indicated that infections in males were more severe and often resulted in fatalities as compared to those in females. This trend was also reflected in the viral titer as measured by the Ct values, where 40% of males had Ct values < 25 (an indicator of high viral titers) compared to 30% of females with Ct values < 25.

**Conclusion:**

Overall, our data indicated that infection rates remained stable throughout the pandemic except for 3rd wave, which showed a higher incidence of infection rate of 4%. Additionally, data showed a positive impact of masking, social distancing, and immunization, as indicated by the shorter window of high infection rates.

## Introduction

The first infection of Severe Acute Respiratory Syndrome Coronavirus 2 (SARS Cov-2) was reported in Wuhan, China, in December 2019 [1]. Soon after the first case, reports of cases started to emerge from all across the globe, culminating in the worst pandemic this century witnessed. World Health Organization (WHO) declared COVID-19 an emergency immediately after deaths started to pour in from other geographic locations around the globe. WHO reported 600 million confirmed cases and 6.5 million deaths between December 2019 and January 2023 [2]. Europe became the first continent to report the highest COVID-19 cases, followed by India in South Asia [3].

The five waves of infections were attributed to five variants of the COVID-19 virus, including the variants of concern and variants of interest reported and classified by WHO and CDC (Centers for Disease Control) [4]. Four variants remained highly pathogenic and caused the highest number of infections. These variants included Alpha (B.1.1.7), which originated from the UK between December 2020 and March 2022; Beta (B.1.351), which originated in South Africa in May 2020; Gamma (p.1) from Brazil in November 2020; and Delta variant (B.1.617.2) was isolated from infections from India in October 2020. Omicron (B.1.1.529), reported from several countries in November 2021 [5], remains the most prevalent circulating variant of concern.

In Pakistan, the National Command and Operation Center (NCOC) reported 1.58 million cases and 30,640 deaths due to COVID-19 [6]. Among the five waves of COVID-19 infections in Pakistan, low death rates were reported during the 1st and 2nd waves due to the well-timed management by the government. Smart and partial lockdowns successfully curbed the transmission of the disease, which led to the implementation the same strategies in the second wave. The first wave caused 6,795 deaths, with 3,321,86 infected and the remaining 632 on ventilators. During the 2nd wave, which lasted till 28 February 2022, 5,79,973 were infected, and 12,860 died [7, 8]. The 3rd wave in Pakistan resulted from the UK variant Alpha (B.1.1.7), which was more lethal than other variants. On average, infections with the alpha variant were associated with 100 deaths per diem in Pakistan. Epidemiological surveys and positivity reports led to a strict lockdown, which was enforced until April 2021 in a total of ten cities in Pakistan, including Bahawalpur, Faisalabad, Hyderabad, Islamabad, Lahore, Multan, Muzaffarabad, Peshawar, Rawalpindi, and Swat. The provincial government observed strict implementation of standard operating procedures [9]. In Summer 2021, the Delta variant reached Pakistan; however, the availability of vaccines and well-managed immunization campaigns reduced its impact [10]. Nevertheless, over 1,245,000 cases were diagnosed during this wave, with approximately 3,000 new cases recorded daily.

The primary method for diagnosing and confirming SARS-CoV-2 in the laboratory relied predominantly on detecting viral RNA in samples collected from the nasopharynx and oropharynx using swabs. The RT-PCR Cycle Threshold (Ct) value, which is inversely proportional to the viral load, was used for reporting results [11]. However, decisions regarding treatment required the inclusion of clinical parameters as well.

In the present study, we intended to observe the trends in COVID-19 infection during the pandemic in the province of Punjab (the most poplated province of the country), Pakistan, using data from a reference lab of Lahore. This study aimed to determine the trends of infections and the association of demographic and anthropometric parameters with positivity rates in the five waves of COVID-19 infection.

## Materials and methods

### Study design, SARS-CoV-2 RNA samples, and cycle threshold (Ct) value collection

#### Data collection process

Data was extracted from the Provincial Public Health Reference Laboratory, Punjab AIDS Control Program, Lahore, designated for COVID-19 diagnostic and screening. The lab bears the highest population load in the province [12] and is a BSL-3 lab under the administrative control of the Punjab AIDS Control Program, Lahore. This laboratory remains open 24/7, and during the COVID-19 pandemic, the lab's testing capacity was increased to 10,000 tests per day. The nasal swabs were obtained from healthcare facilities, communities with high population density or contacts of index cases living close to the index cases.

#### Study design

**Fig. 1:**
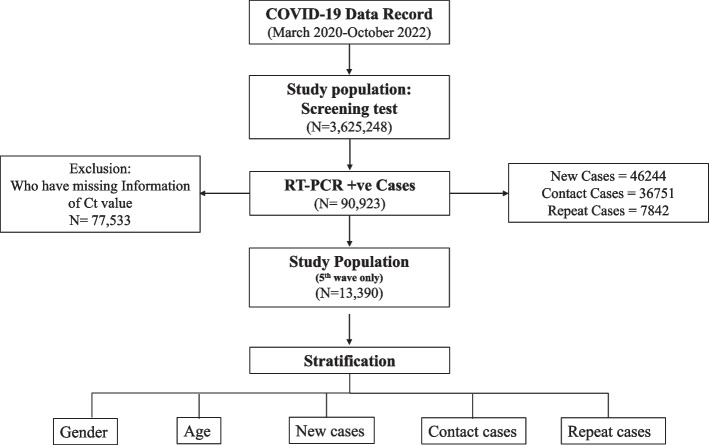
Schematic Presentation of the study Design

This was a retrospective study performed to analyze administrative data answering an important question regarding the effect of different interventions on the transmission of infection. The data was also analyzed by gender, age and viral load. Diagnosed cases in the study were defined as the individuals tested for the presence of the SARS-CoV-2 virus. These cases included symptomatic individuals, close contacts, travellers, healthcare screening workers and an indigenous population. The detailed study design is shown in Fig. 1. The data entered on the dashboard is updated in real-time. The name of the testing laboratory, kit type, result, and result date were recorded in the same dashboard for all PCR tests.

#### Sample processing

Nasopharyngeal swabs were collected from patients and transported to the laboratory at 2-8 °C. In the Laboratory, samples were processed and opened in the Class2 B2 biosafety cabinet in the Biosafety Level 3 section and processed for RNA extraction followed by RT-PCR. In BSL-3, all those samples received from low-infection communities and random sampling areas were pooled and processed for RT-PCR. The detected pool was decoded, and individual samples of that pool were processed to trace the positive sample from that pool. This pooling strategy increased the testing capacity and was successful in resource-limited conditions. The samples received from hospitals and high-infection communities were performed individually for real-time PCR, and all sample results were reported in 24 hours.

#### RNA extraction and RT-PCR

Viral Nucleic Acid Extraction kit (Zybio Inc, China) was used for RNA extraction following the manufacturer's guidelines. Briefly, following RNA extraction from a 200µL aliquot of the primary sample, the elution was carried out using 50 µL of elution buffer. Detection of SARS-CoV-2 was conducted using a SARS-CoV-2 Nucleic Acid Detection Kit following the manufacturer's guidelines. This kit used specific primers and probes targeting the E, N, and RdRP genes of SARS-CoV-2. In addition, primers and probes for the human GAPDH gene were used as an internal control to monitor the entire process [13]. The results were reported according to the previously described criteria [14]. Different SARS-CoV-2 RT PCR Test Kits were employed for amplification. For the N Gene, E Gene, ORF1ab, and RNase P gene, a positive result is reported if Ct equals 36 or as per kit literature accompanied by sigmoid amplification curves for all genes, indicating a positive sample. A sample was considered negative if Ct value was greater than 36 or if there is no Ct value. Furthermore, if all three genes (N Gene, E Gene, and ORF1ab) exhibited positivity, the sample was declared positive. It is noteworthy that the N gene of SARS-CoV-2 serves as the primary positive component in the samples

### Statistical analysis

The data for quantitative variables was expressed as mean ± SD, and the data for qualitative variables was expressed as percentages. Data was checked for normality using Shapiro-Wilk normality test. The data was not normally distributed so, the mean values and percentages were analyzed using the Mann-Whitney U-test and one-way ANOVA to compare differences between two or more groups. All analyses used Graph Pad Prism (Version 8.0, Graph Pad) and SPSS (Version 25.0, IBM).

## Results

### Epidemiological data of COVID-19 in Pakistan

**Fig. 2:**
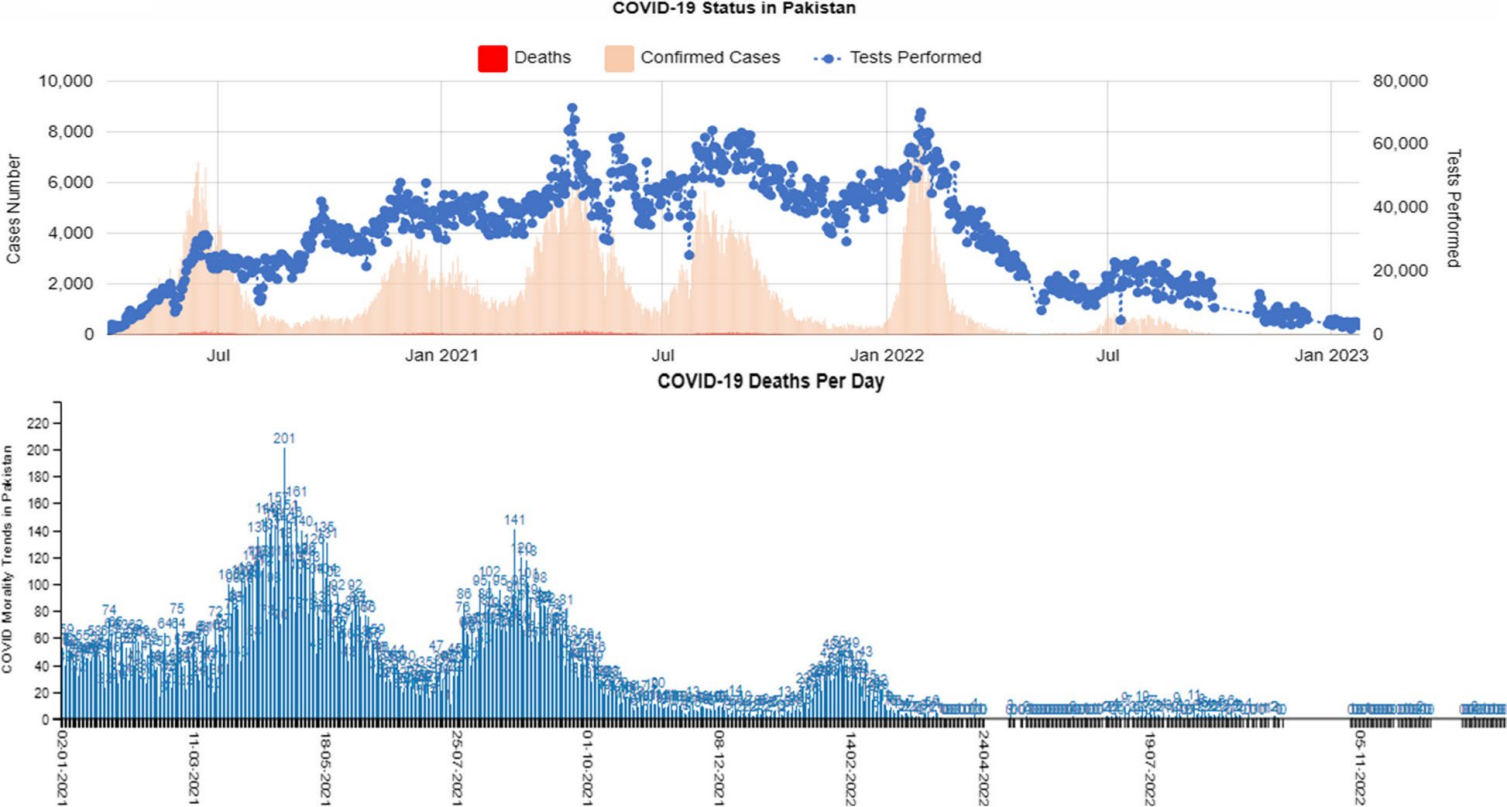
COVID-19 cases reported from Pakistan from March 2020 to Dec 2022

**Fig. 3:**
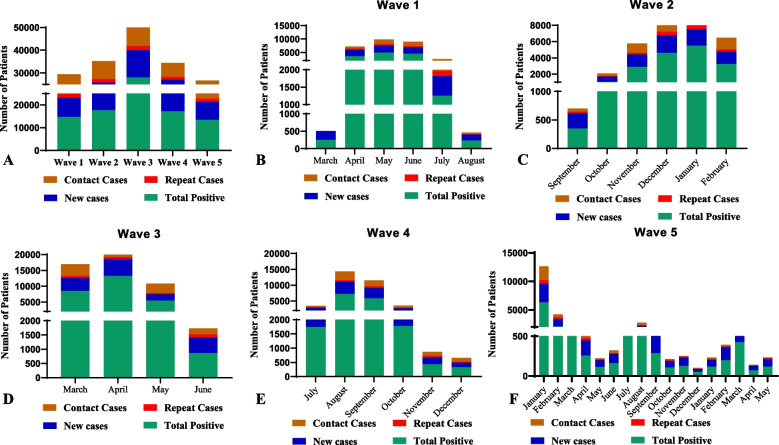
Wave-wise distribution of COVID-19 cases. **A** Accumulative cases of COVID-19, **B** Wave 1 cases, **C** Wave 2 cases, **D** Wave 3 cases, **E** Wave 4 cases, **F** Wave 5 cases

During the five waves of the pandemic, Pakistan reported 1.58 million cases, 1.54 million recoveries, and 30,656 deaths with > 80% vaccination coverage (Fig. 2). Province-wise data showed that Punjab, the most populous province, ranked second in COVID-19 cases with 0.52 million cases, after Sindh. In this study, from March 2020 to May 2023, 36,25248 cases were screened for COVID-19, and 90,923 (2.5%) were confirmed positive cases by RT-PCR, accounting for 5.69% of the cases nationwide. Among the 90,923 positive cases, 50.86% (n = 46244) were new cases (tested for the first time), 40.41% (*n* = 36751) were the contact cases traced from the newly identified cases, and 8.62% (*n* = 7842) were positive among repeat cases (Fig. 3A). The presence of repeat cases suggests that some individuals may experience multiple bouts of infection, emphasizing the potential for reinfection or prolonged viral shedding or to check the severity of infection as suggested by physician. Positivity was detected in a significant number of newly diagnosed cases (< 0.0001).

### Disease transmission dynamics by waves of infection

We measured transmission dynamics over three years to determine the spread of infection. Our results indicated that the dynamic changed over time, as evidenced by the time it took to attain the peak and the wave's duration. The 1^st^ wave of infection (March 2020 – August 2020) lasted 150 days, and the infection peak was attained in 80 days. In 1^st^ wave, 34,998 cases were screened, and the positivity rate was 4.20%. Among all cases that tested positive, the frequency of new, repeat, and contact cases was 56.38% (*n* = 8303), 12.44% (*n* = 1832), and 31.17% (n = 4591), respectively (Fig. 3B). The 2^nd^ wave (September 2020 – February 2021) was similar to the first one; however, it lasted for 165 days before developing into the 3^rd^ wave. In the 2^nd^ wave, 75,0957 cases were screened, and the positivity rate was 2.34%. Among all cases that tested positive, the frequency of new, repeat, and contact cases was 46.39% (*n* = 8164), 8.49% (*n* = 1495), and 45.10% (*n* = 70938), respectively (Fig. 3C). The peak of 3^rd^ wave (March 2021 – June 2021) occurred in 60 days, a much shorter period than the first two waves. The infection lasted 120 days, 60,6889 cases were screened, and the positivity rate was 4.6%. Among all cases that tested positive, the frequency of new, repeat, and contact cases was 42.91% (*n* = 12014), 6.36% (*n* = 1781), and 50.71% (14197), respectively (Fig. 3D). The 4^th^ wave (July 2021 – December 2021) peaked in less than 80 days. The peak was obtained in 55 days, and the infection lasted 160 days. In the 4^th^ wave, 82,2813 cases were screened, and the positivity rate was 2.0%. Among all cases that tested positive, the frequency of new, repeat, and contact cases was 56.37% (*n* = 9769), 7.41% (*n* = 1276), and 35.81% (*n* = 6173), respectively (Fig. 3E). While the infection started to decline in 120 days, a new variant led to the 5^th^ wave of infection (January 2022). The most interesting observation during the 5^th^ wave was the attainment of the peak in a very short period. The infection peak was attained in 38 days, noticeably less than the time taken by the previous waves to attain a peak in infection. In the 5^th^ wave, 112,5230 cases were screened, and the positivity rate was 1.19%. Among all cases that tested positive, the frequency of new, repeat, and contact cases were 58.99% (*n* =7894),10.88% (*n* = 1458), and 29.46% (*n* = 3946), respectively (Fig. 3F). In conclusion, the maximum positivity rate of new cases was 12.3% in 3^rd^ wave, after which it decreased in the subsequent waves (Table 1).

**Table 1:** Wave wise distribution of New, contact and repeat cases tested positive

**COVID-19 Waves**	**Time Period**	**Total Screened Cases**	**Total +ve Cases**	**RT-PCR +ve Cases**			***p*** **-value**
				**New cases** **n (%)**	**Repeat Cases** **n (%)**	**Contact Cases** **n (%)**	
Wave 1	Mar-20	3290	252 (7.65)	250 (99.20)	2 (0.79)	0 (0)	<0.0001
	Apr-20	19804	3597 (18.16)	2374 (65.99)	488 (13.56)	735 (20.43)	<0.0001
	May-20	67779	4900 (7.22)	2584 (52.73)	653 (13.32)	1663 (33.93)	<0.0001
	Jun-20	99666	4494 (4.50)	2359 (52.49)	534 (11.88)	1601 (35.62)	<0.0001
	Jul-20	86854	1253 (1.44)	562 (44.85)	143 (11.41)	548 (43.73)	<0.0001
	Aug-20	72555	230 (0.31)	174 (75.65)	12 (5.21)	44 (19.13)	<0.0001
**Total of wave 1**		349948	14726 (4.20)	8303 (56.38)	1832 (12.44)	4591 (31.17)	<0.0001
Wave 2	Sep-20	105479	351 (0.33)	266 (75.78)	28 (7.97)	57 (16.23)	<0.0001
	Oct-20	97042	1035 (1.06)	645 (62.31)	84 (8.11)	306 (29.56)	<0.0001
	Nov-20	153461	2885 (1.87)	1532 (53.10)	196 (6.79)	1157 (40.10)	<0.0001
	Dec-20	144270	4602 (3.18)	2182 (47.41)	432 (9.38)	1988 (43.19)	<0.0001
	Jan-21	146219	5485 (3.75)	2012 (36.68)	500 (9.11)	2973 (54.20)	<0.0001
	Feb-21	104486	3239 (3.09)	1527 (47.14)	255 (7.87)	1457 (44.98)	<0.0001
**Total of wave 2**		750957	17597 (2.34)	8164 (46.39)	1495 (8.49)	7938 (45.10)	
Wave 3	Mar-21	206775	8477 (4.09)	4077 (48.09)	644 (7.59)	3756 (44.30)	<0.0001
	Apr-21	20326	13244 (65.15)	5355 (40.43)	653 (4.93)	7236 (54.63)	<0.0001
	May-21	194582	5407 (2.77)	2032 (37.58)	383 (7.08)	2992 (55.33)	<0.0001
	Jun-21	185206	864 (0.46)	550 (63.65)	101 (11.68)	213 (24.65)	<0.0001
**Total of wave 3**		606899	27992 (4.61)	12014 (42.91)	1781 (6.36)	14197 (50.71)	
Wave 4	Jul-21	163128	1740 (1.06)	1107 (63.62)	137 (7.87)	496 (28.50)	<0.0001
	Aug-21	163128	7169 (4.39)	3903 (54.44)	490 (6.83)	2776 (38.72)	<0.0001
	Sep-21	145006	5767 (3.97)	3392 (58.81)	428 (7.42)	1947 (33.76)	<0.0001
	Oct-21	146150	1776 (1.21)	964 (54.27)	145 (8.16)	667 (37.55)	<0.0001
	Nov-21	92712	435 (0.46)	241 (55.40)	50 (11.49)	144 (33.10)	<0.0001
	Dec-21	112689	331 (0.29)	162 (48.94)	26 (7.85)	143 (43.20)	<0.0001
**Total of wave 4**		822813	17218 (2.09)	9769 (56.37)	1276 (7.41)	6173 (35.85)	
Wave 5	Jan-22	231883	6311 (2.72)	3230 (51.18)	652 (10.33)	2429 (38.48)	<0.0001
	Feb-22	205545	2106 (1.02)	1278 (60.68)	343 (16.28)	485 (23.02)	<0.0001
	Mar-22	147712	511 (0.34)	329 (64.38)	85 (16.63)	97 (18.98)	<0.0001
	Apr-22	73008	256 (0.35)	191 (74.60)	29 (11.32)	36 (14.06)	<0.0001
	May-22	9876	111 (1.12)	85 (76.57)	13 (11.71)	13 (11.71)	<0.0001
	Jun-22	27686	160 (0.57)	115 (71.87)	11 (6.87)	34 (21.25)	<0.0001
	Jul-22	32242	957 (2.96)	582 (60.81)	67 (7.00)	308 (32.18)	<0.0001
	Aug-22	71997	1370 (1.90)	840 (61.31)	138 (10.07)	392 (28.61)	<0.0001
	Sep-22	97042	285 (0.29)	220 (77.19)	25 (8.77)	40 (14.03)	<0.0001
	Oct-22	37813	119 (0.31)	88 (73.94)	16 (13.4)	15 (12.60)	<0.0001
	Nov-22	40398	125 (0.30)	107 (85.60)	10 (8.00)	7 (5.60)	<0.0001
	Dec-22	35739	51 (0.14)	35 (68.62)	3 (5.88)	13 (25.49)	<0.0001
	Jan-23	31691	117 (0.36)	86 (48.58)	9 (7.69)	22 (18.80)	<0.0001
	Feb-23	26400	196 (0.74)	166 (84.69)	8 (4.08)	22 (11.22)	<0.0001
	Mar-23	28609	424 (1.48)	390 (91.98)	10 (2.35)	24 (5.66)	<0.0001
	Apr-23	12880	69 (0.53)	58 (84.05)	7 (10.14)	4 (5.79)	<0.0001
	May-23	14709	116 (0.78)	94 (81.03)	17 (14.6)	5 (4.31)	<0.0001
**Total of wave 5**		1125230	13390 (1.19)	7894 (58.99)	1458 (10.88)	3946 (29.46)	<0.0001
**Grand Total**		3625248	90923 (2.50)	46244 (50.86)	7842 (8.62)	36751 (40.41)	<0.0001

### Gender and age-based distribution of COVID-19 cases

**Fig. 4:**
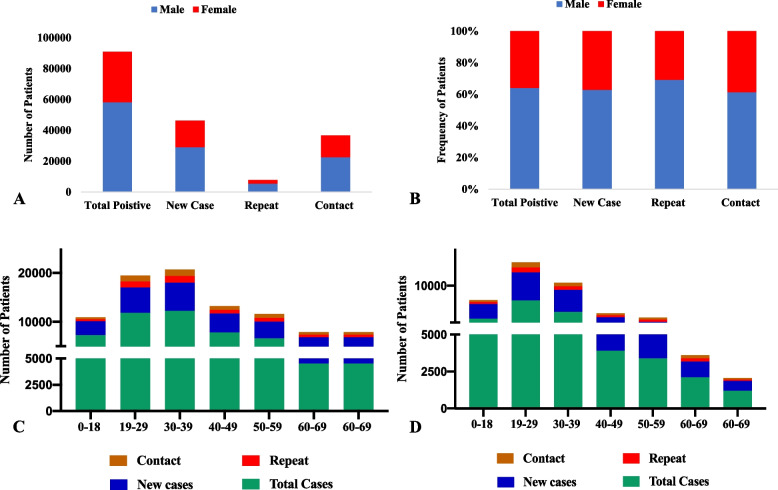
**A** Number of COVID-19 cases across both genders in all waves. **B** Frequency of male and female COVID-19 cases in all waves. **C** Distribution of male COVID-19 cases across different age groups across all waves. **D** Distribution of female COVID-19 cases across different age groups across all waves.

Data based on gender showed that during the pandemic period, the positivity rate among males was 64.03% (*n* = 58218), whereas 35.97% (*n* = 32,705) of females tested positive during the pandemic (Table 2). The proportion of male COVID-19 cases was significantly higher than that of females (*p* < 0.0001; Fig. 4A and B). When stratified by age, a maximum number of cases (*n* = 12,237, 65.52%) were diagnosed in the age group of 30 − 39 years in males (Fig 4C), while in females, the age group of 19 − 29 years (*n* = 7992, 40.34%) was more dominant (Fig. 4D). Overall, in all age groups, the frequency of male patients was significantly higher than that of female patients (*p*-value < 0.0001).

**Table 2:** Distribution of COVID-19 wave across gender and age through the COVID-19 pandemic period

	**Total Poistive** **N (%)**	**New Case** **N (%)**	**Repeat** **N (%)**	**Contact** **N (%)**	***p*** **-value**
**Gender**					
Male	58218 (64.03)	29080 (62.93)	5434 (9.31)	22582 (39.19)	<0.0001
Female	32705 (35.97)	17164 (37.06)	2408 (7.49)	14169 (44.13)	<0.0001
**Total**	**90923**	**46244 (43.91)**	**7842 (8.61)**	**36751 (40.96)**	**<0.0001**
**Age**					
0-18 Y (M)	7271 (56.79)	2832 (38.94)	410 (5.63)	410 (55.24)	<0.0001
0-18 Y (F)	5531 (43.20)	1991 (35.99)	268 (4.84)	268 (59.10)	
19-29 Y (M)	11819 (59.65)	5227 (44.22)	1208 (10.22)	1208 (44.79)	<0.0001
19-29 Y (F)	7992 (40.34)	3786 (47.37)	684 (8.55)	684 (43.98)	
30-39 Y (M)	12237 (65.52)	5774 (47.18)	1345 (10.99)	1345 (41.17)	<0.0001
30-39 Y (F)	6439 (34.47)	2979 (46.26)	500 (7.76)	500 (45.83)	
40-49 Y (M)	7834 (66.74)	3842 (49.04)	757 (9.66)	757 (40.71)	<0.0001
40-49 Y (F)	3904 (33.25)	1809 (46.33)	283 (7.24)	283 (46.36)	
50-59 Y (M)	6634 (66.19)	3381 (50.96)	796 (11.99)	796 (36.08)	<0.0001
50-59 Y (F)	3388 (33.80)	1702 (50.23)	306 (9.03)	306 (40.64)	
60-69 Y(M)	4511 (68.16)	2311 (51.23)	522 (11.57)	522 (36.13)	<0.0001
60-69 Y(F)	2107 (31.83)	1058 (50.21)	216 (10.25)	216 (39.01)	
>69 Y (M)	3097 (72.20)	1763 (56.92)	311 (10.04)	311 (32.80)	<0.0001
>69 Y (F)	1192 (27.79)	667 (55.95)	102 (8.55)	102 (35.40)

### Gender and age-based Ct distribution in 5^th^ wave among COVID-19 cases

**Fig. 5:**
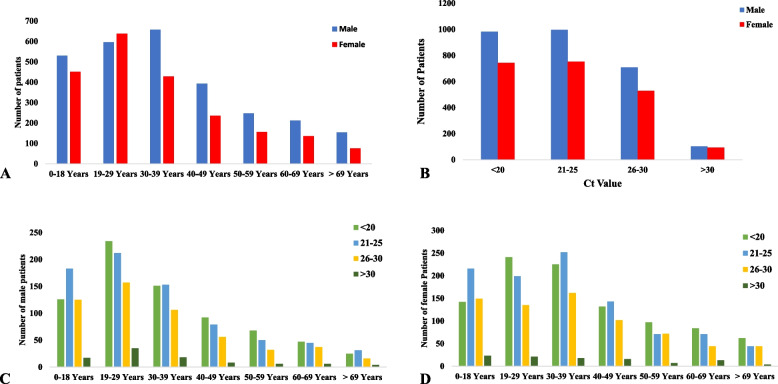
**A** Gender-wise distribution among COVOD-19 cases in 5^th^ wave across different age groups. **B** Gender-wise RT-PCR Ct value distribution among COVOD-19 cases in 5^th^ wave. **C** RT-PCR Ct value distribution among male COVOD-19 cases in 5^th^ wave across different age groups. **D** RT-PCR Ct value distribution among female COVOD-19 cases in 5^th^ wave across different age groups

**Fig. 6:**
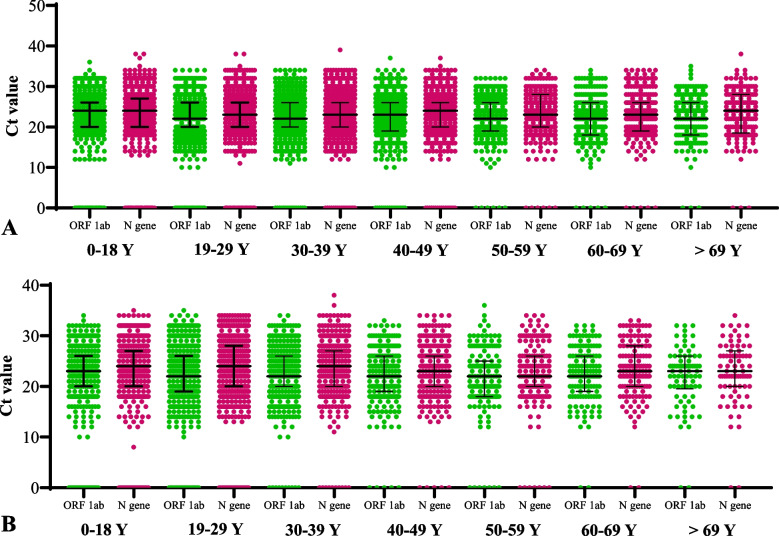
**A** Distribution of Ct value for ORF ad N gene across different age groups among male cases. **B** Distribution of Ct value for ORF ad N gene across different age groups among female COVID-19 cases in 5^th^ wave

Next, we determined the viral titer among positive cases. The viral titer was measured for the 5^th^ wave of the pandemic. The distribution of COVID-19 cases for the 5^th^ wave, predominantly caused by Omicron, is given in Table 3. The gender distribution showed that 56% (*n* = 7502) of cases were male, while 44% (*n* = 5888) were female. The data stratification by age showed that the maximum number of patients was reported from the age group of 19 − 39 years (49%). More females were reported from the age group of 19 − 29 years, while the males were prevalent in the age group of 30 − 39 years (Fig. 5A). Further stratification of the Ct values showed that 35.21% (*n*=2726) of the cases had Ct values < 20, while 35.50% (*n* = 2899) had Ct values between 21 and 25 for both genders (Table 4, Fig. 5B). When stratified by age and gender, 4.77% of females had CT values < 20 for the age group of 19-29 years (Fig. 5C), while 4.58% of males in the age group of 29-39 years had severe infections, as indicated by the lower Ct value (Fig. 5D). The Ct values for ORF and N genes were taken from RT-PCR for the 5^th^ wave and compared using one-way ANOVA. The data showed no significant difference between the ORF and N genes among male and female patients. In addition, no significant difference was observed between the ORF and N genes in any age group among male and female patients (Fig. 6A and B).

**Table 3:** Distribution of COVID-19 cases of 5^th^ wave across gender and age

**Characteristics**	**Total** **N (%)**	**New Case** **N (%)**	**Contact** **N (%)**	**Repeat** **N (%)**	***p*-value**
**Gender**					
Male	7502 (56.19)	4254 (53.57)	2277 (57.58)	837 (58.08)	<0.0001
Female	5888 (43.80)	3638(46.43)	1684 (42.41)	606 (41.92)	<0.0001
**Total**	**13390**	**7892**	**3937**	**1434**	
**Age**					
0-18 Years	2099 (15.89)	1371 (17.70)	530 (13.46)	220 (15.31)	<0.0001
19-29 Years	3878 (28.61)	2390 (29.57)	1082 (27.48)	469 (32.70)	<0.0001
30-39 Years	3533 (26.11)	1990 (250.5)	956 (24.28)	402 (28.03)	<0.0001
40-49 Years	1856 (14.05)	827 (10.68)	514 (13.05)	162 (11.29)	<0.0001
50-59 Years	874 (6.61)	662 (8.55)	438 (11.11)	80 (5.70)	<0.0001
60-69 Years	689 (5.21)	388 (5.01)	250 (6.35)	67 (4.67)	<0.0001
	461 (3.49)	264 (3.40)	167 (4.24)	34 (2.37)	<0.0001
**Total**	**13390**	**7742**	**3937**	**1434**

**Table 4:** RT-PCR Ct value distribution of COVID-19 cases of 5^th^ wave across gender and age

	**< 20 Ct (%)**	**21-25 Ct (%)**	**26-30 Ct (%)**	**> 30 Ct (%)**
Male	1483 (20.03)	1749 (20.29)	1208 (14.43)	130 (2.08)
Female	1243 (15.14)	1150 (15.34)	829 (10.78)	100 (1.92)
**Total**	**2726 (35.21)**	**2899 (35.50)**	**2037 (26.31)**	**230 (2.97)**
0-18 Y (M)	242 (2.89)	316 (4.40)	249 (3.04)	33 (0.47)
0-18 Y (F)	226 (2.57)	283 (3.73)	225 (2.55)	27 (0.35)
19-29 Y (M)	491(2.91)	399 (4.05)	235 (2.75)	31 (0.43)
19-29 Y (F)	484 (4.77)	462 (4.32)	257 (3.20)	39 (0.71)
30-39 Y (M)	325 (4.58)	452 (5.13)	262 (3.30)	18 (0.37)
30-39 Y (F)	251(3.08)	253 (3.12)	206 (2.16)	18 (0.37)
40-49 Y (M)	232 (2.69)	243 (2.91)	102 (2.08)	16 (0.33)
40-49 Y (F)	92 (1.87)	99 (1.61)	56 (1.14)	8 (0.16)
50-59 Y (M)	97 (1.98)	89 (1.45)	72 (1.47)	7 (0.14)
50-59 Y (F)	68 (1.39)	70 (1.02)	32 (0.65)	6 (0.12)
60-69 Y (M)	84 (1.71)	71 (1.45)	44 (0.90)	13 (0.26)
60-69 Y (F)	47 (0.96)	45 (0.92)	37 (0.75)	6 (0.12)
> 69 Y (M)	62 (1.26)	44 (0.90)	44 (0.90)	4 (0.08)
> 69 Y (F)	25 (0.51)	31 (0.63)	16 (0.33)	4 (0.08)

## Discussion

The work presented in this manuscript provides trends in infection in the 5^th^ wave, which was predominantly by the Omicron variant. The wave was associated with increased transmission rates. The Ct data indicated that younger individuals, irrespective of gender, had lower Ct values, indicative of higher viral burden which is likely to be associated with severe infections. Additionally, the disease severity was derived from total no. of cases tested, tested positive and negative. The compasrison of all waves showed that 5^th^ wave has highest cases that were tested positive which is indirectly related to the transmission and incident rate.

A significant difference was observed in positivity rates and transmission dynamics of infection over the five waves. Our findings are in line with the global ternd which showed that different variants have a distinct global spatiotemporal pattern, explaning the occurrence of the five waves in the pandemic. Moreover,different variants demonstrated a distint pattern of transmission, in that Omicron variant indicated better transmissibility in comparison to all previous variants, underscoring the importance of monitoring of any new variants to prevent further transmission [15, 16].

In this study, among the 525,376 confirmed cases, there were 439 reported deaths, including 126 critical cases and 6,507 recoveries. The fatality rate was 1.8%, while the recovery rate was 27%. The incidence of community transmission was reported to be as high as 91% [17]. The elevated rates of prevalence and fatalities in Punjab may be associated with asymptomatic transmission and with the initial untraceable spread of the virus across various districts. The phenomenon was observed globally, where overall asymptomatic transmission accounted for an overall 20% of infection. Epidemiological estimates and mathematical modles demonstrated a 15% transmission in family clusters and 20.5% transmission among adults in general from asymptomatic contact [18]. Further contributing to the infection rate was the rate of mobility. Lahore remains the largest municipal locality in Punjab, followed by Faisalabad, Sialkot and Sargodha. The city is also equipped with state-of-the-art diagnostic facilities. Therefore, a significant number of patients were brought into Lahore from the periphery, thus adding to the number of positive cases.

Increasing age increases the likelihood of hospitalization and death. High-quality evidence shows an age-related risk increase of 5.7% for in-hospital mortality, 7.4% for case mortality and 3.4% for hospitalization [19]. No discernible elevated risk was associated with age for admission to the intensive care unit or intubation. Additionally, a specific age group was not associated with disease severity and mortality [20].

It has been reported that males were at a higher risk of infection, hospitalization, disease severity, and mortality [21]. Several hypotheses, including the possibility of androgen-driven pathogenesis, the potential effect of estrogen in females, testosterone deficiency leading to an inflammatory response, and the notion of an inborn error in cytokine immunity, have been proposed to explain this difference between the two genders [22]. However, additional research is needed to explore these possibilities. The cause is likely multifactorial, with these different hypotheses potentially sharing some common features [23]. Males and people ≥ 70 years of age have been reported to be more susceptible to infection and severe disease [24]. Adolescents are believed to share a comparable susceptibility to infection with adults, while children exhibit a lower susceptibility. Nevertheless, the data for this study presents conflicting findings, and a more comprehensive understanding of the relationship between age and vulnerability to infection requires additional research [25, 26]. However, children are not at a higher risk for developing severe disease [27]. Compared to wild-type viruses, variants have the potential to spread more efficiently and quickly among young children, although there has been a reduction in hospitalization rates [28, 29].

Global COVID-19 data analysis indicates a higher incidence of COVID-19 infection in men than as compared to women [30]. Additionally, a compromised immune system significantly heightens the susceptibility to COVID-19, particularly among the elderly, increasing the likelihood of hospitalization due to virus-related complications. Nevertheless, several studies conducted in Pakistan have presented a paradoxical trend, where the highest number of COVID-19 cases are found in the age groups of 20–29 years and 30–39 years, while the elderly, who are generally more susceptible due to weakened immunity and health issues, have lower infection rates [7, 17, 31]. This apparent discrepancy can be better understood by examining Pakistan's social and demographic structure. According to data from the United Nations, only 4% of Pakistan's population is above 65 years old, with an average population of 22 years. This contrasts sharply with countries heavily impacted by the virus, where older and less healthy individuals are more likely to experience severe consequences due to their weakened immune systems [32].

The epidemiology and trends in spread of infection in Pakistani community can be further explained by the fact that during COVID-19 pandemic, Pakistan, like many other countries, implemented various public health measures. Partial and full lockdowns were imposed in various regions to limit mobility and reduce the spread of the virus. Social distancing measures were put in place together with international and domestic travel restrictions. s. Wearing masks in public places and on public transport was encouraged and, in some cases, made mandatory. In the 2^nd^ wave, in addition to previous restrictions, the government and health authorities launched public awareness campaigns to promote wearing masks, hand hygiene, and social distancing. Increased testing and contact tracing efforts were undertaken to identify and isolate cases promptly. Vaccination efforts began in early 2021 during the 3^rd^ wave, initially targeting healthcare workers and elderly populations. In the 4^th^ wave, concerns about the Delta variant led to increased monitoring of international travellers. Efforts were made to accelerate vaccination campaigns to target a broader population. In response to the emergence of the Omicron variant in the 5^th^ wave, stricter international travel restrictions and monitoring of travellers from affected areas were enforced. Practices including increased testing and timely isolation of cases were emphasized. The government considered administering booster doses to enhance immunity, particularly for those who had received their primary vaccination.

Furthermore, we analyzed the Ct values of COVID-19 cases in particular in the 5^th^ wave were predominantly by the Omicron variant, which was associated with an increased transmission rates. The Ct data indicated that younger individuals, irrespective of gender, had lower Ct values, indicative of severity of infection. The significance of low Ct values lies in their correlation with increased transmission rates. A lower Ct value signifies a higher concentration of the virus in the patient's sample, suggesting a more robust and infectious viral presence. Individuals with lower Ct values may experience more severe symptoms, potentially leading to increased respiratory activities that release a greater number of viral particles into the surrounding environment. Consequently, these factors contribute to the efficiency and persistence of virus transmission.

In conclusion, our observations revealed a higher prevalence of COVID-19 among males, primarily because male family members often work outside the home and have more community interactions than females. Additionally, we noted that individuals between 19 and 39 years were more susceptible to infection. Previous reports have shown that a significant proportion of young adults were affected in most districts of Punjab [33].

### Limitations of the study

Limitations of our study are as follows: First, there is the unavailability of data on clinical symptoms and outcomes of the tested cases. The Ct values were only available for the 5^th^ wave, which made comparing each variant's severity and transmission dynamics across all the waves difficult.Second, due to the unavailability of mobility data, we can only hypothesize that the increased positivity rates were because of paties arriving in Lahore from different parts of Punjab. However, for final analysis, the availability of mobility data is critical. Third, longitudinal data on viral laod was not avialbale due to which the exact rate of viral replication, the duration of shedding, and the potential for transmission could not be accurately determined. Finally, the data used in this study was only taken from one laboratory.

## Data Availability

The datasets used or analysed during the current study are available from the corresponding author upon reasonable request.
